# Chimeric antigen receptor macrophages target and resorb amyloid plaques

**DOI:** 10.1172/jci.insight.175015

**Published:** 2024-03-22

**Authors:** Alexander B. Kim, Qingli Xiao, Ping Yan, Qiuyun Pan, Gaurav Pandey, Susie Grathwohl, Ernesto Gonzales, Isabella Xu, Yoonho Cho, Hans Haecker, Slava Epelman, Abhinav Diwan, Jin-Moo Lee, Carl J. DeSelm

**Affiliations:** 1 Department of Radiation Oncology,; 2 Bursky Center for Human Immunology and Immunotherapy,; 3 Department of Neurology, and; 4 Hope Center for Neurological Disorders, Washington University School of Medicine, St. Louis, Missouri, USA.; 5 Department of Pathology, University of Utah, Salt Lake City, Utah, USA.; 6 Department of Medicine, Division of Cardiology, Peter Munk Cardiac Centre, University Health Network, University of Toronto, Toronto, Ontario, Canada.; 7 Departments of Medicine, Cell Biology and Physiology, Obstetrics and Gynecology, Washington University School of Medicine, St. Louis, Missouri, USA.; 8 Medicine Service, St. Louis VA Medical Center, St. Louis, Missouri, USA.

**Keywords:** Aging, Therapeutics, Alzheimer disease, Immunotherapy, Macrophages

## Abstract

Substantial evidence suggests a role for immunotherapy in treating Alzheimer’s disease (AD). While the precise pathophysiology of AD is incompletely understood, clinical trials of antibodies targeting aggregated forms of β amyloid (Aβ) have shown that reducing amyloid plaques can mitigate cognitive decline in patients with early-stage AD. Here, we describe what we believe to be a novel approach to target and degrade amyloid plaques by genetically engineering macrophages to express an Aβ-targeting chimeric antigen receptor (CAR-Ms). When injected intrahippocampally, first-generation CAR-Ms have limited persistence and fail to significantly reduce plaque load, which led us to engineer next-generation CAR-Ms that secrete M-CSF and self-maintain without exogenous cytokines. Cytokine secreting “reinforced CAR-Ms” have greater survival in the brain niche and significantly reduce plaque load locally in vivo. These findings support CAR-Ms as a platform to rationally target, resorb, and degrade pathogenic material that accumulates with age, as exemplified by targeting Aβ in AD.

## Abstract 2

Macrophages expressing a chimeric antigen receptor directed to beta amyloid can target and degrade amyloid plaques in vivo in a mouse model of Alzheimer's disease.

## Graphical abstract

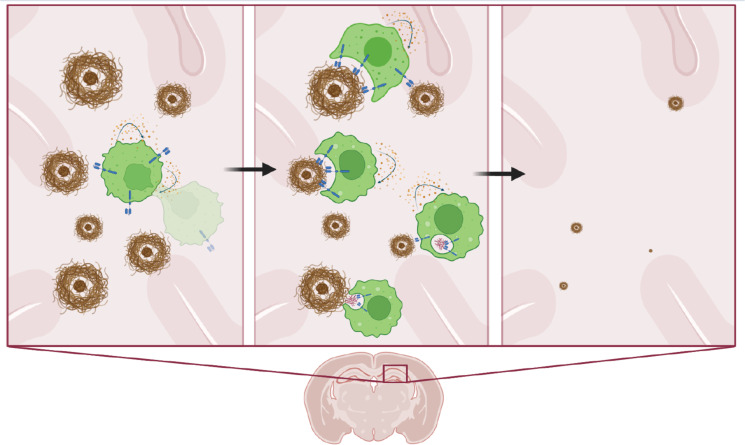

## Introduction

Chimeric antigen receptor (CAR) cellular therapies endow specialized immune cells with the ability to both target a specific antigen and induce a desired cellular function in response to that antigen. Recent breakthroughs in liquid cancer treatment brought on by the advent of CAR T cells (1–5) inspire considerations that CAR cellular therapy may have broader roles in medicine beyond cancer. Indeed, the idea of a living drug that can respond in more sophisticated ways than a single small molecule is appealing for diseases that have thus far evaded conventional approaches. Beyond cancer, Alzheimer’s disease (AD) is one such disease that is both growing in prevalence and is treatable but not curable, despite a large number of mouse and human trials with small molecules, antibodies, and targeted therapies (6, 7).

β Amyloid (Aβ) plaque deposition is hypothesized to be a key initial trigger for AD pathophysiology (8). Antibodies targeting Aβ have recently been shown to reduce amyloid plaque load and mitigate cognitive decline in patients with AD (7, 9), but they have dose-limiting side effects such as amyloid-related imaging abnormalities (ARIA) (10). CAR macrophages (CAR-Ms) have been reported to phagocytose tumor cells in an antigen-specific manner (11) and are currently in a cancer clinical trial (NCT04660929; https://www.clinicaltrials.gov/study/NCT04660929?cond=NCT04660929&rank=1); their use has not yet been extended to noncancerous conditions. Our previous work showed that peripheral monocytes/macrophages are recruited to amyloid plaques in the APP/PS1 transgenic mouse model of AD, where they attenuate amyloid plaque load (12). We hypothesized that enhancing these cells’ capacity to resorb plaque may yield further benefits and wondered if introducing a CAR into macrophages that targets Aβ (using the FDA-approved aducanumab single-chain variable fragment [scFv]) (Supplemental Figure 1A; supplemental material available online with this article; https://doi.org/10.1172/jci.insight.175015DS1) and contains the phagocytic common γ chain of the Fc receptor (FcRγ) as an intracellular signaling domain (13–15) would yield effective Aβ endocytosis in vitro and in vivo. We find that this first-generation CAR-M resorbs soluble Aβ and amyloid plaque in vitro and resorbs and degrades Aβ plaques from APP/PS1 brain slices ex vivo but has limited survival in vivo and fails to significantly reduce local plaque load. Because persistence in the local brain niche appeared to be a problem, we engineered a next-generation, “reinforced CAR-M” that secretes M-CSF to facilitate its own survival, and we found that it significantly expands in the brain microenvironment after microglia depletion with the CSF-1 inhibitor PLX5622. Furthermore, M-CSF reinforced that CAR-Ms significantly resorb plaques locally in the hippocampi of aged APP/PS1 mice compared with control M-CSF–secreting CAR-Ms targeting an irrelevant antigen. These studies demonstrate that targeted CAR-Ms may be utilized to resorb Aβ in vivo, that CAR-Ms rationally engineered to produce cytokines is a strategy to improve their efficacy, and more broadly, that living drugs in the form of cell therapy may be considered in future treatment approaches for AD.

## Results

### Aβ CAR-Ms significantly bind and resorb Aβ in culture and adopt a unique CAR and signaling-induced phenotype.

**Figure 1:**
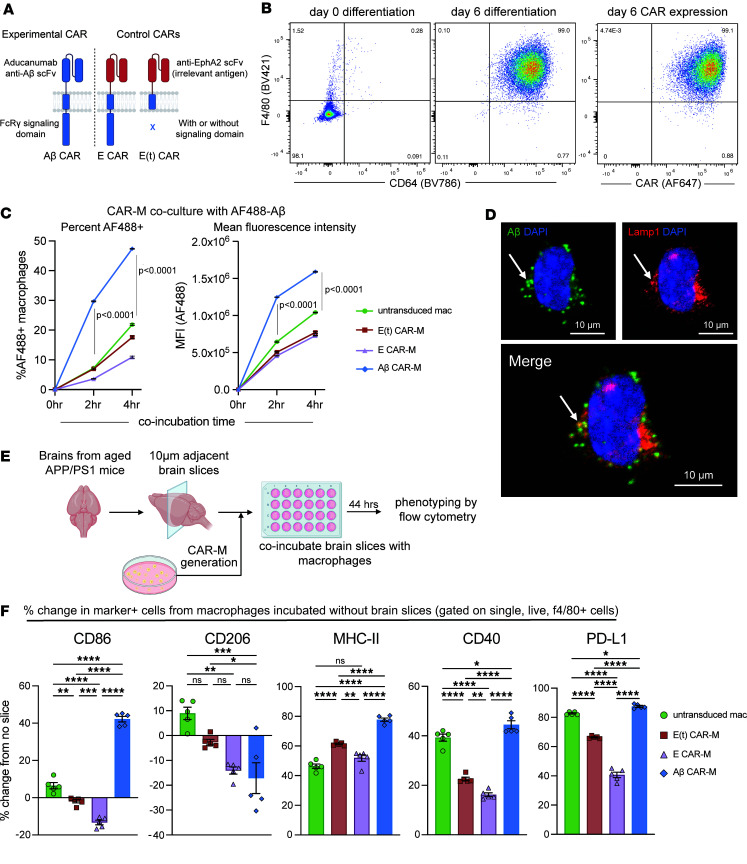
Generation, validation, and phenotype of an aducanumab-based Aβ CAR-M. (**A**) Schematic diagram of Aβ CAR and control CAR constructs. scFv, single-chain variable fragment; E CAR, EphA2 scFv CAR; E(t) CAR, EphA2 truncated CAR. (**B**) Representative FACS plots of (left) F4/80 and CD64 surface expression on HoxB8 cells at day 0 and day 6 of differentiation with M-CSF and (right) surface CAR expression on cells at day 6 of differentiation. (**C**) In vitro uptake of Alexa Flour 488 fluorescent tagged Aβ (1–42 aa) by untransduced, control, or Aβ CAR-Ms after 2 or 4 hours of coincubation, depicted as percent uptake (left) or geometric mean fluorescence intensity (MFI, right). (**D**) Representative immunofluorescence microscopy images of Aβ CAR-Ms incubated with AF-488–tagged Aβ (1–42 aa) for 4 hours and stained for Lamp1. Scale bars: 10 μm. (**E**) Schematic of flow-based phenotyping of untransduced macrophages, control CAR-Ms, or Aβ CAR-Ms coincubated with amyloid-laden brain slices from aged APP/PS1 mice for 44 hours prior to flow cytometry analysis. (**F**) Percent change in the stated marker after coincubation of untransduced, control, or Aβ CAR-Ms on amyloid-laden brain slices from APP/PS1 mice from the expression of the same markers on the same cells incubated without brain slices. Data are represented as mean ± SEM from *n* = 2 independent experiments with 3–5 technical replicates for each condition (**C**) and 5 technical replicates (**F**). Statistical significance was calculated with a repeated measures 2-way ANOVA (**C**) or 1-way ANOVA (**F**) with Tukey’s multiple-comparison tests. For **F**, *P < 0.05, **P < 0.01, ***P < 0.001, ****P < 0.0001.

To reproducibly manufacture CAR-Ms that express the CAR to a high level and avoid the effects of variable transduction efficiency between experiments, we retrovirally introduced control or experimental CAR constructs (Supplemental Figure 1B) into the HoxB8 cell line, which was generated by transducing mouse bone marrow cells with the previously described estrogen-responsive HoxB8 construct that sustains the cells in an undifferentiated state in the presence of estrogen while maintaining their ability to differentiate into macrophages in the presence of M-CSF without estrogen (16). To discriminate between the effects of transduction with a CAR of similar structure but no target binding or signaling, as well as tonic signaling but no binding, we generated 2 additional control CARs composed of (a) a nonspecific extracellular scFv targeting EphA2 (not expressed on normal brain tissue; refs. 17, 18) with an intracellular FcRγ signaling domain, termed “E CAR-M,” and (b) the same EphA2 scFv truncated after the transmembrane domain, thus containing no intracellular signaling domain, termed “E(t) CAR-M” (Figure 1A).

We first confirmed surface CAR expression on macrophages after transduction and differentiation of HoxB8 cells with M-CSF (Figure 1B). HoxB8 cells expressing control (E CAR-M and E[t] CAR-M) or Aβ-targeting CARs to a matched level were sorted, and stocks were generated to ensure consistent CAR expression in all experiments (Supplemental Figure 1C). To test CAR function, untransduced macrophages, E CAR-Ms, E(t) CAR-Ms, or Aβ CAR-Ms were cocultured with Alexa Flour 488–labeled (AF-488–labeled) Aβ (1–42 aa) and analyzed by flow cytometry, which demonstrated a significant time and Aβ CAR–dependent increase in the percentage of cells that took up Aβ as well as the amount of Aβ taken up by each cell (Figure 1C). We confirmed Aβ uptake into Aβ CAR-Ms using confocal microscopy, which localized Aβ to punctate intracellular vesicles and the Lamp1-expressing lysosomal compartment (Figure 1D).

Our Aβ CAR construct contains an extracellular binding domain derived from the aducanumab antibody, which is known to preferentially bind aggregated forms of Aβ, including oligomers and fibrils (19). Thus, we wanted to test whether Aβ CAR-Ms could also take up Aβ oligomers and fibrils. To measure Aβ uptake, we labeled monomeric, oligomeric, or fibril forms of Aβ with a fluorescent dye, AF-488, and cocultured controls or Aβ CAR-Ms with these 3 forms of Aβ for 2 or 4 hours. Aβ uptake was assessed by measuring AF-488 fluorescence in the macrophages by flow cytometry, which confirmed that Aβ CAR-Ms more effectively took up all 3 forms of Aβ compared with untransduced or control CAR-Ms (Supplemental Figure 2, A and B).

To assess the effect of CAR signaling on macrophage phenotype using a more physiologically relevant target, we cultured control macrophages and CAR-Ms on amyloid-laden brain slices from aged APP/PS1 mice. We then examined downstream functional molecules putatively modulated by Fc receptor signaling (20, 21) before and after exposure to Aβ-containing brain slices using flow cytometry (Figure 1, E and F, and Supplemental Figure 1D). Compared with controls, Aβ CAR-Ms upregulated CD86, MHC-II, CD40, and PD-L1 and downregulated CD206 after coincubation with amyloid-laden brain slices. An effect of transduction alone is observed by comparing untransduced macrophages to E(t) CAR-Ms, and an effect of tonic signaling alone is seen by comparing E(t) CAR-Ms and E CAR-Ms. These in vitro experiments demonstrate that the CAR-Ms undergo Aβ CAR-dependent, target-induced phenotype changes.

Because the CAR exerts effects through both target binding and intracellular signaling, we chose to continue the rest of our studies with experimental Aβ CAR-Ms and control E(t) CAR-Ms, which will be henceforth referred to as “control CAR-M.”

### Aβ CAR-Ms significantly reduce plaque load on brain slices ex vivo and resorb plaques of all sizes.

**Figure 2:**
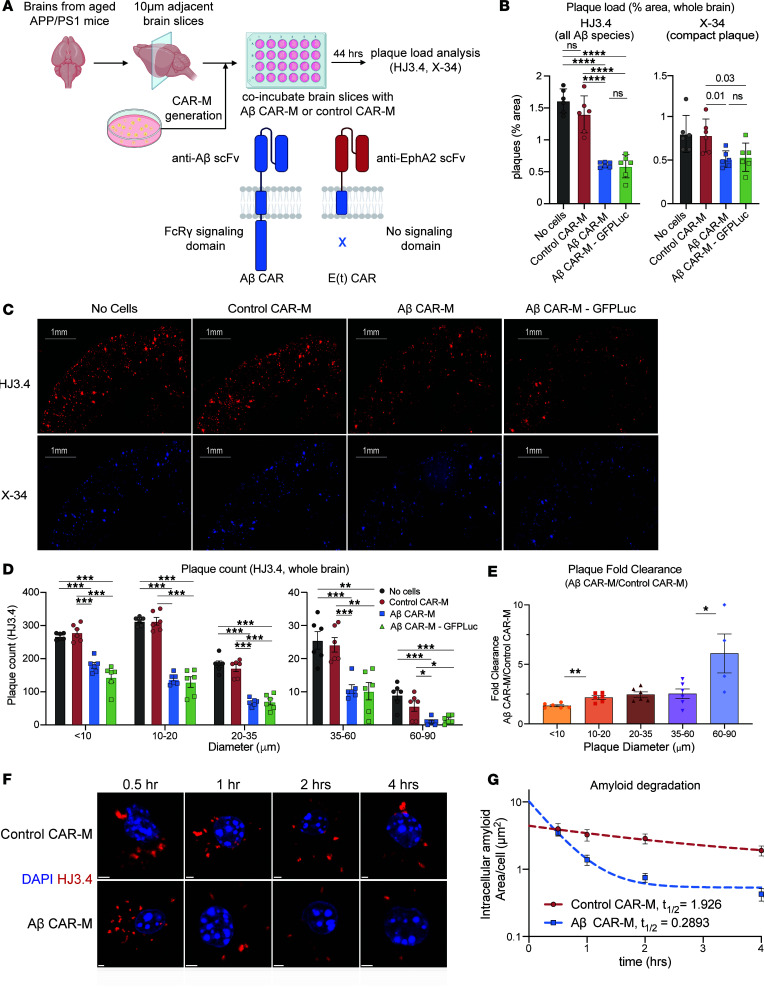
Aβ CAR-Ms resorb amyloid plaques of various sizes on brain slices from aged APP/PS1 mice ex vivo and degrade amyloid more efficiently than control macrophages. (**A**) Schematic of ex vivo assessment of amyloid plaque phagocytosis. Adjacent brain slices from aged APP/PS1 mice were coincubated with Aβ CAR-M or control CAR-Ms for 44 hours, and plaque load was assessed with HJ3.4 or X-34 immunostaining. (**B**–**E**) Assessment of plaque load (**B** and **C**), plaque count (**D**), and plaque fold clearance of Aβ CAR-M over control CAR-Ms (**E**) on APP/PS1 brain slices after coincubation with no cells, control CAR-M, or Aβ CAR-M with or without GFP-Luciferase. Data shown as mean ± SEM from *n* = 5–6 independent experiments with 5–6 technical replicates each. Scale bars: 1 mm. Statistical significance was calculated with 1-way ANOVA with Tukey’s multiple-comparison test (**C** [HJ3.4], **D**) or unpaired 2-tailed *t* tests (**C** [X-34], **E**). *P < 0.05, **P < 0.01, ***P < 0.001, ****P < 0.0001. In **E**, “60–90” only contains 4 data points due to a lack of plaques this size in some samples. (**F**) Representative immunofluorescence microscopy images of individual amyloid-laden control or Aβ CAR-Ms after removal from brain slices from aged APP/PS1 mice for the indicated amount of time and stained with HJ3.4 to visualize intracellular amyloid over time. Scale bars: 2 μm. (**G**) Quantification of intracellular amyloid in control of Aβ CAR-Ms cultured on brain slices from aged APP/PS1 mice for the indicated amount of time. Data are shown as mean ± SEM from 18 technical replicates. Each condition was fitted to a 1-phase exponential decay function to derive the indicated half-lives.

To assess the ability of Aβ CAR-Ms to resorb plaques that were deposited in vivo, we again coincubated control or Aβ CAR-Ms on brain slices from aged APP/PS1 mice (Figure 2A) and, this time, also introduced GFP-Luciferase (GFP-Luc) into the cells in preparation for in vivo monitoring. Quantification of HJ3.4– and X-34–stained plaques, which recognizes all forms of Aβ (22) and fibrillar compact plaques (23), respectively, revealed a significant reduction in plaque burden in Aβ CAR-M–treated slices over control CAR-M slices ex vivo, with no detrimental effect of additional GFP-Luc expression (Figure 2, B and C). Thus, all subsequent studies used GFP-Luc expressing control and Aβ CAR-Ms.

Further analysis of the sizes of plaques being resorbed on APP/PS1 brain slices showed that Aβ CAR-Ms effectively resorb plaques of all sizes (Figure 2D). In addition, when we assessed fold change in plaque clearance of Aβ-targeted versus control CAR-Ms, Aβ-targeted CAR-Ms resorbed larger plaques of > 10 microns relatively more effectively than smaller plaques (Figure 2E). This, as well as direct immunohistological visualization of plaque-CAR-M interactions (Supplemental Figure 3, E and F), suggests that plaque is likely resorbed in pieces rather than being taken up by whole-plaque phagocytosis.

### Aβ CAR-Ms degrade intracellular amyloid.

In addition to taking up Aβ, we determined whether Aβ CAR-Ms could more efficiently degrade the resorbed intracellular Aβ. CAR-Ms were incubated with amyloid-laden brain slices for 4 hours; cells were then removed from the slices and grown in culture. At various time points thereafter, cells were fixed and immunostained with HJ3.4 to track intracellular amyloid over time (Figure 2F). We quantified the area of Aβ immunostaining per cell and plotted it over time. Intriguingly, although Aβ CAR-Ms take up more Aβ, they also degrade it more efficiently compared with control CAR-Ms (Figure 2G). The half-life of intracellular amyloid in control CAR-Ms was 1.926 hours, while the half-life in Aβ CAR-Ms was only 0.2893 hours. Thus, Aβ CAR-Ms not only take up more Aβ than control CAR-Ms, but they also degrade intracellular Aβ faster.

### First-generation Aβ CAR-Ms find plaque in vivo but have limited expansion and survival.

Next, we wanted to test the ability of Aβ CAR-Ms to target and reduce amyloid plaques in vivo. Given the importance of preconditioning in CAR T cell therapy (24–26) and microglia engraftment studies (27), we preconditioned mice with a nonablative dose of Busulfan (28, 29) for 2 days to deplete endogenous microglia prior to intrahippocampal injection of PBS or Aβ CAR-Ms (Supplemental Figure 3A). Since it was uncertain if the CAR-Ms would spread within the brain, we chose to inject one hippocampus with cells and the contralateral hippocampus with PBS (vehicle).

Engraftment of CAR-Ms was assessed using bioluminescence imaging (BLI) in WT and APP/PS1 mice, which showed that, in APP/PS1 mice, CAR-Ms survived and modestly expanded for the first 10–12 days but subsequently diminished to baseline by day 14 (Supplemental Figure 3, B and C). Plaque load was quantified on day 14 in a circular region of interest (ROI) surrounding the injection site, centered around residual GFP signal in the hippocampal region (Supplemental Figure 3D). Uniform circle sizes were used across all slices. Aβ CAR-Ms recognized and bound Aβ plaques (Supplemental Figure 3E); however, no significant difference in plaque load was observed between PBS and Aβ CAR-M–treated hemispheres (Supplemental Figure 3F). Because the injected CAR-Ms appeared to stay relatively localized to the injection site (Supplemental Figure 3D), for subsequent studies, we decided to inject the left hippocampus of each mouse with control CAR-Ms and the right hippocampus with Aβ CAR-Ms. This bilateral injection paradigm also controls for intermouse heterogeneity in plaque load.

### Reinforced CAR-Ms expressing M-CSF have enhanced survival and locally reduce hippocampal plaque load in vivo.

**Figure 3:**
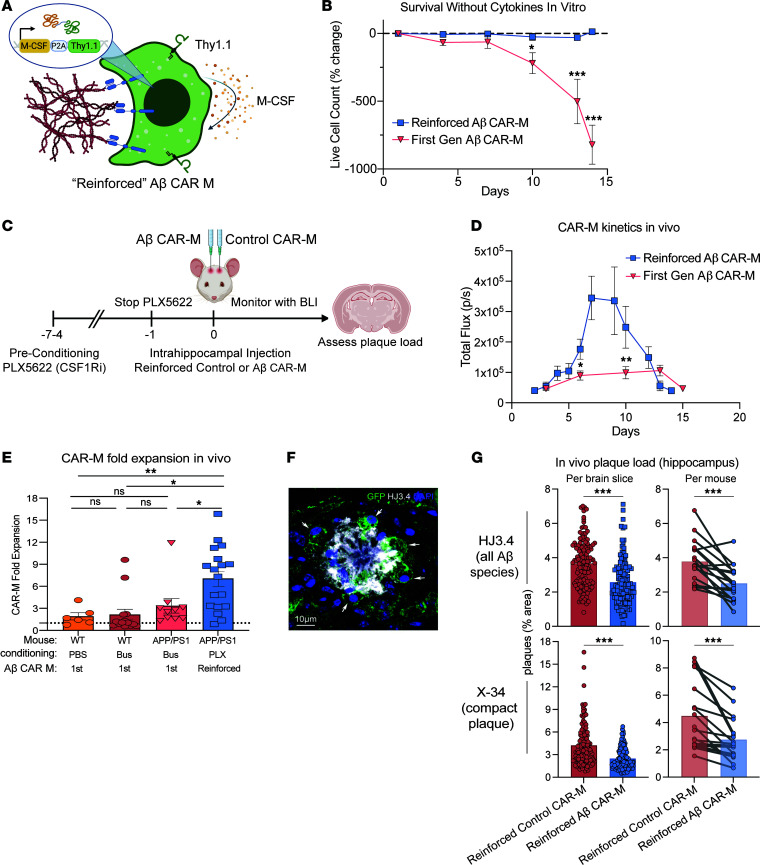
M-CSF reinforced Aβ CAR-Ms have improved survival and reduce plaque load in vivo. (**A**) Schematic of M-CSF expressing construct retrovirally introduced into control and Aβ CAR-M, which contains the M-CSF gene followed by a P2A cleavage sequence and Thy1.1. (**B**) Percent change in live cell count of first-generation Aβ CAR-Ms and M-CSF reinforced Aβ CAR-Ms upon removal of M-CSF from the culture medium in vitro*,* determined by flow cytometry staining with Zombie NIR live/dead staining. Cells were differentiated for 6 days in M-CSF to become mature macrophages, prior to M-CSF removal. Statistical significance was calculated with an unpaired 2-tailed *t* test. (**C**) Schematic of PLX5622 preconditioning and intrahippocampal injection of M-CSF reinforced Aβ CAR-Ms. (**D**) Total flux determined by noninvasive bioluminescence imaging (BLI) tracking first-generation Aβ CAR-M kinetics in vivo compared with M-CSF reinforced Aβ CAR-M kinetics after intrahippocampal injection. “Days” indicates days after intrahippocampal injection. *n* = 10–18 mice per group. Statistical significance was calculated with unpaired 2-tailed *t* tests. (**E**) Fold-expansion of CAR-Ms from the first day of BLI after intrahippocampal injection of cells to the day of maximum total flux measured by BLI. *n* = 6–18 mice per group. Statistical significance was calculated with 1-way ANOVA with Tukey’s multiple-comparison test. (**F**) Representative immunofluorescence microscopy image of M-CSF reinforced Aβ CAR-Ms binding to amyloid plaque in vivo. Scale bar: 10 μm. (**G**) Assessment of plaque load after intrahippocampal injection of M-CSF reinforced control CAR-M or M-CSF reinforced Aβ CAR-M in *n* = 12 (14-month-old female APP/PS1 mice) and *n* = 6 (13-month-old male APP/PS1 mice). Arrows point to GFP^+^ CAR-Ms bound to and phagocytosing amyloid plaque. Mice were sacrificed on day 12 or 13 after intrahippocampal injection, and brain tissue was sectioned and stained with HJ3.4 and X-34 to assess plaque load. Data are shown as mean ± SEM. Statistical significance was calculated with unpaired 2-tailed *t* tests for brain slice data and paired 2-tailed *t* tests for per mouse data. For **B**–**D**, *P < 0.05, **P < 0.01, ***P < 0.001.

Due to the lack of survival, persistence, and efficacy of first-generation CAR-Ms in vivo, we developed reinforced CAR-Ms that secrete M-CSF to improve persistence and efficacy in vivo (Figure 3A and Supplemental Figure 4A). While first-generation CAR-Ms nearly completely die within 2 weeks without exogenous cytokine, M-CSF reinforced CAR-Ms persist at a stable level in vitro in the absence of exogenous cytokine for at least a month (Figure 3B and Supplemental Figure 4B).

Reinforced Aβ CAR-Ms express F4/80, CD64, and CCR2 and lack expression of Ly6C and CD62L, suggesting that they become mature macrophages after M-CSF differentiation (Supplemental Figure 4C). To further characterize these cells, we stained M-CSF reinforced and first-generation Aβ CAR-Ms with Cell Trace Violet (CTV) to measure their proliferation in vitro in the absence of exogenous M-CSF. A small proliferating population within the floating cells and a larger nonproliferating population of attached cells exist in both cultures. However, in M-CSF reinforced CAR-Ms, there is a significantly larger proliferating population (Supplemental Figure 4D). For all experimental studies, we only use attached cells with less proliferative capacity but more homogenous differentiation.

To assess in vivo survival and expansion of reinforced CAR-Ms, we injected M-CSF reinforced control and Aβ CAR-Ms in opposite hippocampi of aged APP/PS1 mice. To deplete endogenous microglia while sparing nonmyeloid cells with a potentially more clinically translatable drug (30, 31), we subjected mice to 4–7 days of preconditioning with the CSF-1 inhibitor PLX5622 (Figure 3C). BLI showed M-CSF reinforced CAR-Ms rapidly expanded in the first week within the brain, then plateaued and rapidly contracted (Figure 3D and Supplemental Figure 5A). Although we saw only modest microglial depletion with this preconditioning regimen compared with PBS or low-dose Busulfan preconditioning (Supplemental Figure 5C), reinforced CAR-Ms reached significantly greater numbers than first-generation CAR-Ms (Figure 3D), expanding in all but 1 of 18 mice with an average 7-fold expansion in vivo (Figure 3E).

Histologic analysis demonstrated that Aβ CAR-Ms recognized plaque in vivo and demonstrated plaque phagocytosis (Figure 3F). Quantification of plaque load in the previously defined ROI around the injection site (Supplemental Figure 6A) revealed a significant reduction in diffuse and compact plaques after M-CSF reinforced Aβ CAR-M treatment compared with M-CSF reinforced control CAR-M treatment. This result holds whether plaque load is analyzed per brain slice or per individual mouse (Figure 3G). We again observed little migration of CAR-Ms away from the injection site, and analysis of random ROIs in mirror regions of the cortex did not reveal significant changes in plaque load (Supplemental Figure 6, A and B), demonstrating relative uniformity of plaque load across hemispheres and no change in plaque load in regions in which CAR-Ms were not present. These results hold when these data are stratified by sex (Supplemental Figure 6, C and D).

## Discussion

In this study, we show that macrophages expressing an Aβ-targeting CAR take up Aβ peptide in vitro and significantly reduce plaque load on amyloid-laden brain slices ex vivo. We further show that M-CSF reinforced Aβ CAR-Ms significantly reduce diffuse and compact hippocampal plaque load in vivo when injected into the hippocampus of aged APP/PS1 mice after preconditioning with a CSF-1 inhibitor to deplete endogenous microglia.

These results demonstrate that, while macrophages expressing a CAR targeting Aβ can significantly resorb Aβ and amyloid plaques in vitro and ex vivo, achieving significant in vivo plaque resorption required engineering the cells to self-sustain by producing M-CSF for autocrine and/or paracrine stimulation. While M-CSF secretion alone may have achieved some degree of plaque clearance through either microglia stimulation (32) or improving CSF flow dynamics (33), we measured a significant independent CAR target-mediated effect, since our control hemispheres were also treated with M-CSF secreting CAR-Ms but not targeting Aβ. We found that Aβ CAR signaling also significantly changed the phenotype of macrophages upon target binding in vitro, which may itself affect both how the CAR-M functions and how it modulates the local microenvironment. Since engineering M-CSF secretion into the CAR-M had a significant effect, creating additional reinforced CAR-Ms that secrete other microenvironment-modulating cytokines of interest may be another future approach to rationally reshape the local environment.

There are several important limitations to these studies, and clinically relevant technical improvements would be needed for CAR-Ms to access the entire brain in order to have the potential for clinical benefit in treating AD. CAR-Ms injected into the hippocampus of APP/PS1 mice were limited in their survival and migration within the brain niche. We have not examined why the CAR-Ms fail to persist long-term within the brain, but several possibilities exist. Just as microglia have different phenotypes throughout the brain due to differences in local microenvironment (34), fully differentiated CAR-Ms may lack the complete set of receptors necessary to thrive within the brain niche. Even if they can survive within the brain, they may become out-competed by more fit microglia as they recover from preconditioning depletion. Alternatively, the CAR-Ms may be immunologically depleted, due to either MHC expression of foreign antigenic peptides (such as from GFP-Luc; ref. 35, 36) or due to mixed strain immunoreactivity (APP/PS1 mice are on a mixed C57BL/6 × C3H background, since pure C57BL/6 APP/PS1 mice develop seizures at a considerable rate) (37). While the mechanism of failure of long-term CAR-M engraftment into the brain is of interest academically, it is not clear whether achieving long-term engraftment would be desired clinically. The fact that these CAR-Ms are allogeneic and do not persist long-term but still have a significant local effect in vivo is encouraging for the possibility that an allogeneic product may be feasible, if CAR-Ms were to become therapeutically relevant for AD in the future. Next-step studies that improve migration throughout the brain and, ideally, CNS penetration after IV delivery will be important for achieving full clinical potential. Our previous work showed that peripheral monocytes can enter the AD brain in APP/PS1 mice and reduce plaque load in the absence of any intervention (12), suggesting that increasing the number of peripheral monocytes entering the brain, especially Aβ CAR-modified ones, will further reduce plaque load. Enhancing CAR-Ms with factors that promote peripheral myeloid cell entry, adaptation, and migration to the brain niche may represent additional strategies to boost therapeutic potential. Another potential strategy for clinical translation is the delivery of anti–Aβ CAR mRNA with monocyte/macrophage-targeted lipid nanoparticles, as has been done for CAR T cells in the setting of cardiac fibrosis (38). While aducanumab is known to preferentially bind aggregated forms of Aβ (19), we could not directly compare the ability of Aβ CAR-Ms to take up various forms of Aβ because, while we attempted to minimize monomer aggregation during resuspension, we cannot confirm the stability of the monomeric, oligomeric, and fibril Aβ throughout the duration of our phagocytosis assay. Furthermore, the fluorescence labeling may prefer one Aβ species over another.

In several neurological diseases, including AD, the microglial niche is known to undergo phenotypic changes that can be both beneficial and detrimental to disease progression (39). Because of these insights, there have been several efforts to replace dysfunctional microglia with healthy microglia or with peripheral monocytes via microglial or bone marrow transplant after preconditioning with myeloablative chemotherapy or radiation to remove endogenous microglia (27, 40). These studies also show that, while monocytes do not infiltrate the brain parenchyma under homeostatic conditions, monocytes can infiltrate the brain parenchyma in the setting of preconditioning chemotherapy or radiation, which — in addition to depleting endogenous microglia to make room for new cells to engraft — presumably promotes peripheral monocyte infiltration into the brain due to disruption of entry sites into the CNS, including the blood-brain barrier, and promotes increased secretion of cytokines and chemokines that recruit peripheral immune cells (41, 42). In line with these studies, we also observed that preconditioning with agents that reduce endogenous microglia — in our case, nonmyeloablative (low dose) Busulfan (43) — improves engraftment of peripheral myeloid cells in both WT and APP/PS1 mice compared with PBS preconditioning. Better microglial removal has been achieved with myeloablative doses of Busulfan followed by bone marrow transplant (44); however, we intentionally did not pursue fully ablative regimens that have little potential for clinical translation. PLX5622 is reported to robustly deplete microglia as well as all other myeloid cells in other models, if given ad libitum in chow for an extended period (30, 45). In our studies, this more specific microglia-depleting preconditioning regimen was associated with significantly reduced plaque load in the hippocampus of Aβ CAR-M–treated APP/PS1 mice. Since a similar PLX CSF1R inhibitor is currently FDA approved for a rare myeloid-derived tumor and has been well tolerated (46, 47), this may be a more clinically translatable preconditioning regimen. We appreciated only modest microglia reduction after delivering the drug i.p. twice a day for 4 days, and although microglia are expected to quickly recover after discontinuation of PLX5622, we still observed substantial proliferation of M-CSF reinforced CAR-Ms in that setting.

Accumulating evidence suggests that peripheral monocytes/macrophages phagocytose and degrade Aβ plaques more effectively than microglia. Transgenic mice that block peripheral myeloid cell infiltration into the brain parenchyma have increased Aβ plaque load compared with control APP/PS1 mice, suggesting that peripherally derived myeloid cells phagocytose and eliminate plaques better than brain-resident microglia (48). Conversely, the depletion of microglia in APP/PS1 mice led to no change in amyloid plaque count or size that developed over time, again implying that microglia are unable to effectively phagocytose and degrade amyloid plaques, though the ability of peripheral macrophages to reduce amyloid plaque load was not assessed in this study (49). The inability of late stage–disease microglia to control plaque load with AD progression may be attributed to the reduced expression of Aβ binding receptors and Aβ-degrading enzymes as plaque deposition increases with age, and this was shown to lead to decreased phagocytosis and degradation of amyloid material in APP/PS1 mice (50). Similarly, microglia isolated from human AD brains show reduced expression of molecules important for phagocytosis and the recycling of phagocytic receptors (51). Recent reports suggest that microglia may even promote the spread and development of Aβ plaques (52, 53). Peripheral macrophages, however, may also be defective at phagocytosing Aβ plaques in the setting of AD, potentially due to chronic Aβ exposure; it has been shown that monocytes/macrophages from patients with AD were less effective at phagocytosing Aβ compared with cells from age-matched patients without AD (54). Allogenic CAR-M therapy may be a solution to overcome dysfunctional cell-mediated amyloid plaque clearance in AD and provides a theoretical advantage over therapeutic strategies that require functional endogenous cells to remove Aβ.

While we did not perform a direct comparison of Aβ CAR-Ms and anti-Aβ monoclonal antibodies, theoretical advantages of CAR-Ms include (a) their ability to be engineered to express factors that can reshape or reinforce cell phenotype or local environments and (b) constitutive phagocytosis and degradation of plaque material due to endogenous CAR expression that does not rely on antibody encounter or resident microglia for Fc mediated uptake. Furthermore, while endogenous microglia or macrophages may become dysfunctional with age (55), CAR-Ms could theoretically be engineered from allogeneic young healthy donors to overcome functional challenges facing aged cells.

Our findings that Aβ CAR-Ms not only take up more amyloid but also degrade it more quickly and to a greater degree than control macrophages imply that the CAR enhances the resorptive capacity of the cell as well as intracellular processing of resorbed contents. There may be additional functional consequences resulting from retained intracellular amyloid, which remain to be fully examined. Mechanistically, FcRγ signaling has been shown to drive the expression of lysosomal genes involved in degradation and killing of lysosomal cargo (56), suggesting that additional FcRγ signaling from the intracellular signaling domain upon amyloid binding in Aβ CAR-Ms may explain the increased rate and magnitude of amyloid degradation observed. FcRγ signaling can activate lysosomal gene expression changes through the transcription factor TFEB (56), which is also known to enhance Aβ degradation in vivo through lysosomal biogenesis and degradative functions (57, 58).

The ability of CAR-Ms to degrade amyloid rather than redistribute it may also lead to reduced incidence of ARIA compared with antibody therapy. ARIA is thought to occur in the setting of anti-Aβ monoclonal antibody therapy when amyloid-antibody complexes are cleared via perivascular clearance mechanisms and accumulate in perivascular spaces, which can impair further perivascular drainage of these complexes and cause inflammatory reactions that damage the brain vasculature and disrupt the blood-brain barrier, resulting in edema and microhemorrhage (59, 60). Future studies will determine if CAR-Ms can reduce amyloid plaque burden with less incidence of ARIA than anti-Aβ monoclonal antibody therapy.

We acknowledge that this work builds upon the amyloid hypothesis of AD, but the clinical efficacy observed in more recent clinical trial results with additional anti-amyloid antibodies, lecanemab (7) and donanemab (61), suggest that further development of novel anti-amyloid therapies is warranted. Although we use Aβ as the target of these CAR-Ms, theoretically, any pathogenic material may be targeted and degraded by replacing the scFv domain on this CAR backbone. These data support the further development of CAR-M technology beyond cancer and establish CAR-Ms as one additional potential approach in the therapeutic toolbox for AD.

## Methods

### Sex as a biological variable.

Our study examined male and female mice, and similar findings are reported for both sexes.

### Plasmids.

The Aβ CAR construct was generated by adding the aducanumab scFv and the murine Fcγ receptor (mFcγR) extracellular, transmembrane, and intracellular domains to a murine leukemia virus (MuLV) retroviral backbone. The control CAR constructs were generated by adding an anti-EphA2 scFv with or without the FcγR intracellular domain to the retroviral backbone. The GFP-luc construct was made from GFP and F-Luc in a MuLV retroviral backbone. The M-CSF construct was generated by adding Thy1.1 and M-CSF to the retroviral backbone.

### Cell lines.

RD114 (Biovac) and Plat-E (Cell BioLab Inc.) cells were cultured in DMEM (Sigma-Aldrich) with 10% FBS (Atlas Biologicals) and 1% penicillin and streptomycin (Thermo Fisher Scientific). The generation of HoxB8 cells has been previously described (16). Briefly, femoral bone marrow cells from C57BL/6J mice were isolated and cultured in recombinant mouse IL-3, IL-6, and SCF for 2 days. On the third day of culture, cells were cultured in media with FLT3-ligand and infected with retroviral introduction of an MSCV vector containing the mouse Hoxb8 DNA and a human estrogen receptor binding domain with a mutation that prevents physiological concentrations of estrogen from binding. HoxB8 cells were maintained in RPMI media (Thermo Fisher Scientific) with 10% FBS (Atlas Biologicals), 1% penicillin and streptomycin (Thermo Fisher Scientific), 1% sodium pyruvate (Thermo Fisher Scientific), nonessential amino acids (Thermo Fisher Scientific), HEPES (Thermo-Fischer), 2 Mm L-glutamine, 0.1% β-mercaptoethanol (Thermo Fisher Scientific), FLT3-ligand (in-house), and β-estradiol (MilliporeSigma). All the HoxB8 cells were transduced with retroviral vectors obtained from Platinum-E (Plat-E) cell lines. After transduction, FACS was performed to obtain 100% retroviral vector^+^ HoxB8 cells. To induce differentiation, sorted HoxB8 cells were incubated in RPMI media supplemented with 10% M-CSF for 6 days. Macrophages were lifted from cell culture plates for downstream assays with Accutase (Innovative Cell Technologies). All the cell lines were regularly tested for mycoplasma by in-house PCR.

### Cell transfection and transduction.

The RD114 retroviral packaging cell line was seeded to 6-well plates at a concentration of 500,000 cells/well the day before transfection. In total, 2.5 μg of plasmid DNA was mixed with 5 μL polyethyleneimine (PEI) at 1 μg/mL stock solution (Alfa Aesar) and incubated for 15 minutes at room temperature before being added to the RD114 cells. Sixteen to 24 hours later, media were changed and retrovirus was collected for use starting 24 hours later. RD114 virus was used to generate stable virus-producer Plat-E cell lines generated via retroviral transduction with 8 μg/mL polybrene and spinfection at 1,800*g* for 1 hour. Plat-E virus was used to transduce HoxB8 cells, which were transduced with 4 μg/mL polybrene and spinfection at 600*g* for 30 minutes.

### Flow cytometry.

All antibodies were titrated. CAR expression was measured with goat anti–human IgG Alexa Fluor 647 (catalog 156339, Jackson ImmunoResearch), and transduction with the M-CSF construct was measured with anti–Thy1.1 PE (clone OX-7, catalog 202524, BioLegend). M-CSF differentiated macrophages were stained with Fc block and Oligoblock (made in-house), anti–F4/80 BV421 (clone T45-2342, catalog 565411, BD Biosciences), and anti–CD64 BV786 (clone X54-5/7.1, catalog 569507, BD Biosciences). Macrophages incubated on APP/PS1 brain slices were phenotyped by staining with anti–CD86 BV605 (clone GL-1, catalog 105037, BioLegend), anti–MHC-II FITC (clone M5/114.15.2, catalog 107606, BioLegend), anti–PD-L1 BV421 (clone MIH5, catalog 564716, BD Biosciences), anti–CD40 PeCy5 (clone 3/23, catalog 124618, BioLegend), and anti–CD206 BV711 (clone C068C2, catalog 141727, BioLegend). Live/dead staining and the M-CSF CAR-M viability assay was conducted with Zombie NIR Fixable Viabilty Dye (catalog 423106, BioLegend). M-CSF reinforced macrophages were phenotyped with additional markers: anti–F4/80 PerCP-Vio700 (clone REA126, catalog 130-118-466, Miltenyi), anti–CCR2 PE (clone QA18A56, catalog 160105, BioLegend), anti–CD62L BV711 (clone MEL14, catalog 104445, BioLegend), and anti–Ly6C APC(clone HK1.4, catalog 128015, BioLegend). First-generation and M-CSF reinforced CAR-M proliferation was measured by staining the cells with CellTrace Violet, according to manufacterer’s instructions (catalog C34557, Invitrogen). Flow cytometry data were acquired on a Cytek NL-3000, and data were analyzed with FlowJo version 10.8.1 (Tree Star Inc.).

### In vitro Aβ phagocytosis assay.

Untransduced or CAR-Ms were generated by differentiating HoxB8 cells with M-CSF for 6 days and were then seeded in 96-well plates at a concentration of 20,000/well in 200 μL RPMI. In total, 1 μL of either HiLyte Fluor 488–labeled Aβ (1–42 aa) (Anaspec) prepared by reconstituting 0.1 mg in 50 μL of 1% NH_4_OH or AF-488–labeled monomeric, oligomeric, or preformed fibril Aβ protein was added into each well. Monomeric (StressMarq Biosciences, catalog SPR-485), oligomeric (StressMarq Biosciences, catalog SPR-488), and fibril (StressMarq Biosciences, catalog SPR-492) Aβ proteins were labeled with the AF-488 Microscale Protein Labeing Kit (Invitrogen, catalog A30006) according to manufacturer’s instructions and used immediately after labeling. Oligomeric and fibril Aβ were generated by the manufacturer using HFIP-treated and dried Aβ monomers, and they were resuspended according to manufacturer’s instructions to ensure retention of oligomeric or fibril form. Throughout the labeling process and assay set up, peptides were kept on ice whenever possible to minimize aggregation, though this does not exclude the possibility that aggregation occurred in culture.

After 2 and 4 hours of coincubation, the wells were washed with PBS, stained with Zombie NIR Fixable Viability dye and anti-F4/80 BV421, and analyzed by flow cytometry. Some of the cells were plated onto 8-well chamber slides (Ibidi, catalog 80826) for confocal microscopy. Briefly, cells were permeabilized and blocked with 0.3% Triton X-100/3% dry milk in 0.01M PBS for 15 minutes, followed by incubation with primary antibodies overnight at 4°C and fluorescently labeled secondary antibodies at 37°C for 1 hour; primary and secondary antibodies are shown in Supplemental Table 1. After staining, cells were rinsed in PBS and images were acquired on a Nikon AXR laser scanning confocal microscope with a 60× oil immersion objective. Image analysis was conducted using ImageJ (NIH).

### Ex vivo plaque phagocytosis assay, degradation assay, and CAR-M phenotyping.

Twelve- to 14-month-old APP/PS1 mice (B6.Cg-Tg[APPswe,PSEN1dE9]85Dbo/Mmjax, The Jackson Laboratory) were perfused with PBS prior to brain extraction. Brains were frozen on dry ice and then stored at –80°C. Prior to each assay, frozen brains were sectioned into 10 μm slices using a CryoStat and were subsequently placed on poly-D-lysine–coated coverslips. The coverslips were then placed in cell culture plates immersed in culture media.

CAR Hoxb8 cells were induced to differentiate to macrophages in RPMI media with M-CSF for 6 days. On day 6, floating cells were washed away with PBS and adherent CAR-Ms were lifted with Accutase (Innovative Cell Technologies). Brain slices were incubated with 2 × 10^5^ untransduced macrophages, control CAR-M, or Aβ CAR-Ms in 1 mL of differentiation media (RPMI + M-CSF) for 44 hours. Cells were incubated on adjacent brain slices to approximately match plaque load and distribution across conditions. For phenotyping, macrophages were lifted from the brain slices using Trypsin EDTA (0.05%, Thermo Fisher Scientific, catalog 25300054) and stained for flow cytometry as above. For plaque analysis, cells were cultured on adjacent brain slices to approximately match plaque load and distribution. After incubation, brain slices were fixed in 4% PFA for 20 minutes. Slices were then stained with X-34 dye or HJ3.4 antibody. High-resolution images of the slices were taken using a NanoZoomer Digital Scanner (Hamamatsu Photonics). The total area of plaque coverage was measured with ImageJ and expressed as percentage total area. The frequency of size classified plaques was analyzed with ImageJ.

To analyze intracellular plaque degradation, macrophages were lifted from brain slices using Trypsin EDTA and passaged into 8-well chamber slides (Lab-Tek, catalog 154941). After incubation for 0.5 hours, 1 hour, 2 hours, and 4 hours, cells were fixed with 4% PFA and then stained with HJ3.4 antibody and DAPI. Images were acquired using Zeiss LSM 980 Airyscan 2 confocal microscope. Surface area of intracellular plaques and cell counting were analyzed with Imaris 10.0 software and expressed as area per cell.

### Intracranial injection and BLI imaging.

APP/PS1 mice (B6.C3-Tg[APPswe,PSEN1dE9]85Dbo/Mmjax; The Jackson Laboratory, MMRRC stock no. 34928, maintained as C57BL/6 × C3H strain), 12–14 months of age, were preconditioned with either 20 mg/kg Busulfan once a day for 2 days or PLX5622 (HY-114153, MedChemExpress) at 50 mg/kg twice daily for 4–7 days by i.p. injection. Busulfan was dissolved in DMSO (MilliporeSigma) at a concentration of 30 mg/mL and diluted to 3 mg/mL in PBS for injection. PLX5622 was dissolved in DMSO at a concentration of 50 mg/mL and diluted to 5 mg/mL in 20% Kolliphor RH40 (Sigma-Aldrich) in PBS.

Cells were prepared for intracranial injection by incubating CAR HoxB8 cells in 10% M-CSF differentiation media for 6 days. On day 6 after differentiation, the macrophages were lifted with Accutase, washed 3 times with PBS, and loaded into a 5 μL Hamilton syringe at a concentration of 1.5 × 10^5^ cells/μL. A total of 2 μL of Aβ CAR-Ms was injected into the right hippocampus and 2 μL of PBS or control CAR-Ms was injected into the left hippocampus (anteroposterior [AP], –2.0; mediolateral [ML], ±1.6; dorsoventral [DV], –1.5). A total of 200 ng/mL recombinant M-CSF was added to the cell preparations to improve cell viability during injections. BLI was performed every 3–7 days; mouse heads were shaved before imaging. Mice were grouped in 3-day intervals when charting BLI over time.

### IHC and plaque staining for in vivo studies.

Twelve- to 14-month-old male and female APP/PS1 mice were sacrificed on day 14 with Fatal-Plus and perfused with PBS. For microglia-depletion studies (Supplemental Figure 5B), mice were sacrificed on day 4 after preconditioning with PBS, Busulfan, or PLX5622 as above. Brains were removed, fixed for 24 hours in 4% paraformaldehyde fixative in 0.1M phosphate buffer (PB) (pH 7.4), and then transferred to a solution containing 30% sucrose in 0.1M PB until the tissue sank down. The brain was then sectioned (40 μm), and 12 equally spaced sections (80 μm apart) containing the dorsal part of hippocampus were immunostained using antibodies against GFP and HJ3.4 delineated in Supplemental Table 1. For microglia-depletion studies, brain sections were stained with Iba1. Brain sections were permeabilized and blocked with 0.3% Triton X-100/ 3% dry milk in 0.01M PBS for 30 minutes, followed by incubation with primary antibodies overnight at 4°C and fluorescently labeled secondary antibodies at 37°C for 1 hour. Primary and secondary antibodies employed are shown in Supplemental Table 1. For X-34 staining, brain slices were mounted on glass slides. Tissue was permeabilized with 0.25% Triton for 30 minutes and stained with X-34 dissolved in a solution of 40% ethanol in water, pH 10, for 20 minutes. The tissue was then rinsed in distilled water and mounted. For in vivo plaque load studies, 12 female, 14-month-old APP/PS1 mice and 6 male, 13-month-old APP/PS1 mice were used.

### Assessment of microglial depletion with preconditioning.

Images were acquired with a Biotek Cytation 5 (Agilent), and images were analyzed with Biotek Gen5 software. For each mouse, 6 ROIs were captured in each hippocampus at 20× magnification, for a total of 12 ROIs per mouse and 3 mice per group. In each ROI, Iba-1^+^ cells were counted using the following parameters in Gen5: threshold minimum, 4,800; minimum size, 10 μm; maximum size, 60 μm; include primary edge objects; do not split touching objects. This mask was applied using the same settings to all ROIs across conditions to quantify microglia in the hippocampus.

### Quantification of amyloid plaque load.

Images were acquired using a Zeiss Axio Scan Z1 or a Nikon AXR Confocal Microscope. For each mouse, 5–9 sections containing the dorsal hippocampus (spaced 80 μm apart) were analyzed using ImageJ. Circular ROIs with uniform areas of 3,622 μm^2^ were created, centered around the injection site in the hippocampus (residual GFP signal). Plaque load was quantified within these circles using brain sections stained with either HJ3.4 or X-34. Plaque load was expressed as a percentage of area within the ROI. Uniform circle sizes were used across all slices. For controls, circular ROIs in mirror cortical regions were analyzed to ensure the density of plaques was relatively uniform across mirror regions of each hemisphere.

### Statistics.

Statistical analyses were performed in GraphPad Prism version 9.5.1. Results are expressed as mean ± SEM. Statistical differences were assessed with the unpaired 2-tailed Student’s *t* test for comparison of 2 experimental groups or 1- or 2-way ANOVA for 3 or more experimental groups. *P* values from ANOVA with multiple comparisons were generated with Tukey’s multiple-comparison test. A 2-tailed *P* value of less than 0.05 was considered statistically significant.

### Study approval.

All animal studies were approved by the IACUC at Washington University School of Medicine.

### Data availability.

Values for all data points in graphs are reported in the Supporting Data Values file. 

## Author contributions

Conceptualization and methodology were contributed by ABK, QX, PY, QP, AD, JML, and CDJ. Experiments were performed by ABK, QX, PY, QP, GP, IX, and YC. Mouse surgeries were performed by SG and EG. Analysis was contributed by ABK, QX, PY, and QP. Writing of the original draft and figure preparation were contributed by ABK and CJD. Review and editing of the manuscript were contributed by ABK, QX, PY, QP, HH, SE, AD, JML, and CJD. Resources were contributed by HH, AD, JML, and CJD. Supervision and funding were contributed by AD, JML, and CJD.

## Supplementary Material

Supplemental data

Supporting data values

## Version 1 03/22/2024

Electronic publication
