# Supplementary material for: Chimeric antigen receptor macrophages target and resorb amyloid plaques
Source: JCI Insight. 2024 Mar 22;9(6):e175015. doi: 10.1172/jci.insight.175015 (PMC11063938; doi:10.1172/jci.insight.175015)
Supplement: Supplemental data [file jciinsight-9-175015-s055.pdf]

supplemental 1

a

Aducanumab vH:
QVQLVESGGGVVQPGRSLRLSCAASGFAFSSYGMHWVRQAPGKGLEWVAVIWFDGTKKYYTDSVKGRFTISRDNSKNTLYLQMNTLRAEDTAVYY
CARDRGIGARRGPYYMDVWGKGTTVTVSSASTKGPSVFPLAPSSKSTSGGTAALGCLVKDYFPEPVTVSWNSGALTSGVHTFPAVLQSSGLYSLSS
VVTVPSSSLGTQTYICNVNHKPSNTKVDKRVEPKSC

aducanumab vL:
DIQMTQSPSSLSASVGDRVTITCRASQSISSYLNWYQQKPGKAPKLLIYAASSLQSGVPSRFSGSGSGTDFTLTISSLQPEDFATYYCQQSYSTPLTFG
GGTKVEIKRTVAAPSVFIFPPSDEQLKSGTASVVCLLNNFYPREAKVQWKVDNALQSGNSQESVTEQDSKDSTYSLSSTLTLSKADYEKHKVYACEVT
HQGLSSPVTKSFNRGEC

CAR extracellular linker, transmembrane, and intracellular domain derived from the common gamma chain:
LDEPQLCYILDAVLFLYGIVLTLLYCRLKIQVRKAAIASREKADAVYTGLNTRSQETYETLKHEKPPQ

A FLAG tag was placed between the scFv and extracellular linker.

Full CAR sequence (without signal peptide):
QVQLVESGGGVVQPGRSLRLSCAASGFAFSSYGMHWVRQAPGKGLEWVAVIWFDGTKKYYTDSVKGRFTISRDNSKNTLYLQMNTLRAEDTAVYYC
ARDRGIGARRGPYYMDVWGKGTTVTVSSASTKGPSVFPLAPSSKSTSGGTAALGCLVKDYFPEPVTVSWNSGALTSGVHTFPAVLQSSGLYSLSSVV
TVPSSSLGTQTYICNVNHKPSNTKVDKRVEPKSCGGGGSGGGGSGGGGSDIQMTQSPSSLSASVGDRVTITCRASQSISSYLNWYQQKPGKAPKLLI
YAASSLQSGVPSRFSGSGSGTDFTLTISSLQPEDFATYYCQQSYSTPLTFGGGTKVEIKRTVAAPSVFIFPPSDEQLKSGTASVVCLLNNFYPREAKVQ
WKVDNALQSGNSQESVTEQDSKDSTYSLSSTLTLSKADYEKHKVYACEVTHQGLSSPVTKSFNRGECLEDYKDDDDKLDEPQLCYILDAVLFLYGIVLT
LLYCRLKIQVRKAAIASREKADAVYTGLNTRSQETYETLKHEKPPQ

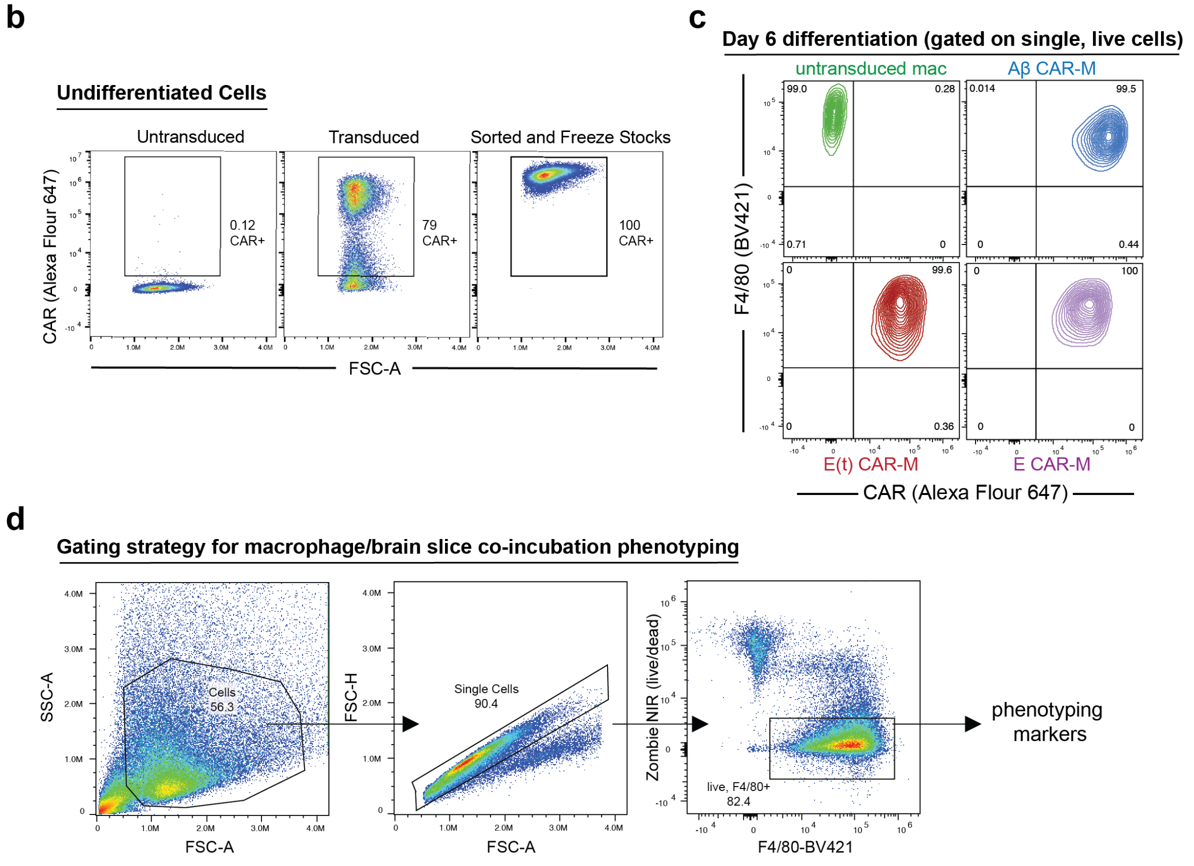

Supplemental Figure 1: Generation, validation, and phenotyping CAR HoxB8 cells.

a) Amino acid sequence of the aducanumab based scFv and rest of the Aβ CAR construct. **b)** Representative FACS plots showing surface expression of the Aβ CAR on untransduced, retrovirally transduced, and transduced and sorted HoxB8 cells used for downstream

experiments. Numbers represent percentage of cells in the indicated gate. Representative of n>3 independent experiments. **c)** Representative FACS plots showing surface expression of F4/80 (y-axis) and control or Aβ CAR (x-axis) on sorted HoxB8 cells differentiated into mature macrophages with M-CSF for 6 days. Representative of n>3 independent experiments. **d)** Gating strategy used for flow cytometry phenotyping of untransduced macrophages, control CAR-Ms, or Aβ CAR-Ms incubated on brain slices from aged APP/PS1 mice. Phenotyping markers were assessed on single, live, f4/80+ cells. SSC-A, side scatter area; FSC-A, forward scatter area; FSC-H, forward scatter height.

supplemental 2

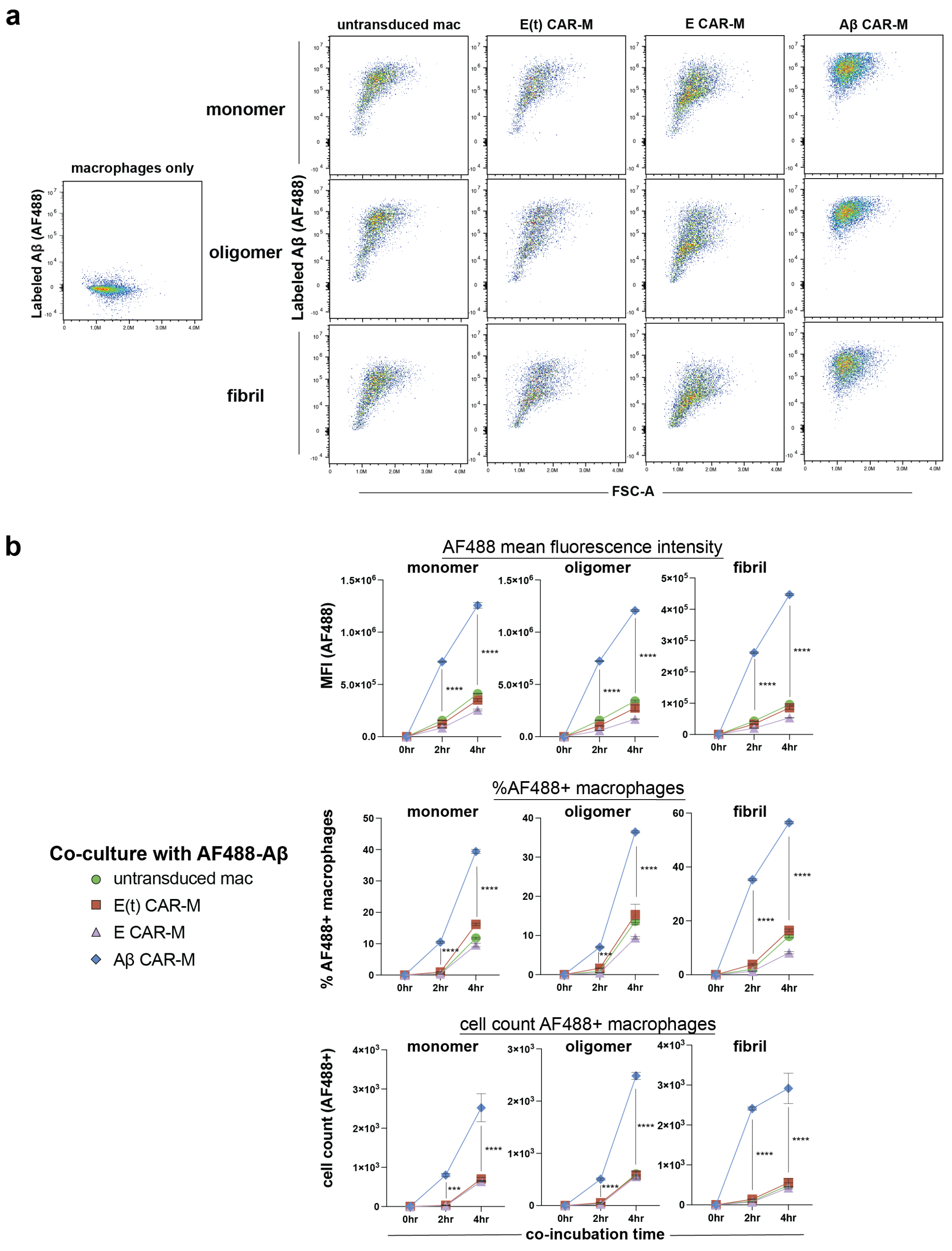

Supplemental Figure 2: An anti-Aβ CAR allows macrophages to take up various forms of Aβ more effectively than control CAR or untransduced macrophages. a) Representative

flow cytometry plots showing uptake of AF488-labeled Aβ in the indicated forms by untransduced, control CAR, or Aβ CAR macrophages after 2 hours of co-culture in vitro (y-axis). Left plot indicates background levels of AF488 fluorescence in a sample that contained macrophages only without AF488-labeled Aβ. FSC-A, forward scatter area. **b)** Quantification of monomeric, oligomeric, or fibril Aβ uptake after the given amount of time, shown as AF488 mean fluorescence intensity (top), the proportion of macrophages that were AF488+ (middle), or the cell count of AF488+ macrophages (bottom). Data are shown as mean ± s.e.m. and is representative of 2 independent experiments with 3 technical replicates per condition per experiment. Statistical significance calculated with 2-way ANOVA with Tukey's multiple comparisons test. $^{*}P < 0.05$, $^{**}P < 0.01$, $^{***}P < 0.001$, $^{****}P < 0.0001$.

supplemental 3

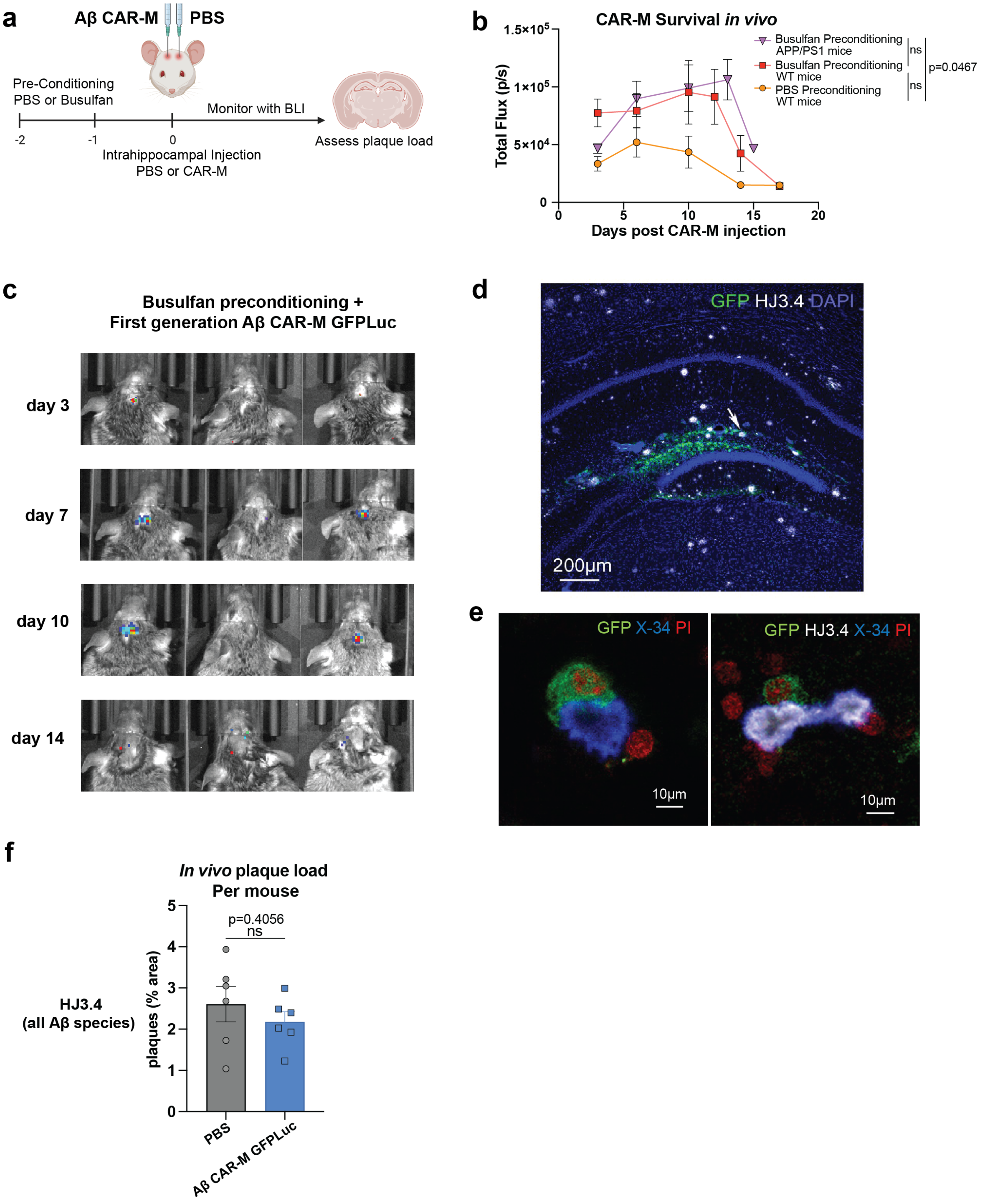

Supplemental Figure 3: First generation Aβ CAR-Ms have limited expansion and survival and fail to reduce plaque load in vivo when administered with Busulfan preconditioning. **a)** Schematic of Busulfan pre-conditioning and intrahippocampal injection of Aβ CAR-Ms. **b)** Non-invasive bioluminescence imaging (BLI) tracking CAR-M persistence after intrahippocampal injection. n=6-14 mice per group. Statistical significance calculated with one-way ANOVA with Tukey's multiple comparisons test. **c)** Representative BLI images following Busulfan preconditioning and intrahippocampal injection of Aβ CAR-Ms. Days indicates days post-intrahippocampal injection. **d)** Representative immunofluorescence microscopy image showing GFP+ CAR-Ms localized to the hippocampus 14 days after intrahippocampal injection. **e)** Representative immunofluorescence microscopy images of Aβ CAR-Ms binding to amyloid plaque in vivo. **f)** Assessment of plaque load after intrahippocampal injection of PBS or Aβ CAR-Ms in n=6 aged APP/PS1 mice. Mice were sacrificed on day 14 post intrahippocampal injection and brain tissue was sectioned and stained with HJ3.4 to assess plaque load. Data shown as mean ± s.e.m. Statistical significance was calculated with unpaired t-tests.

supplemental 4

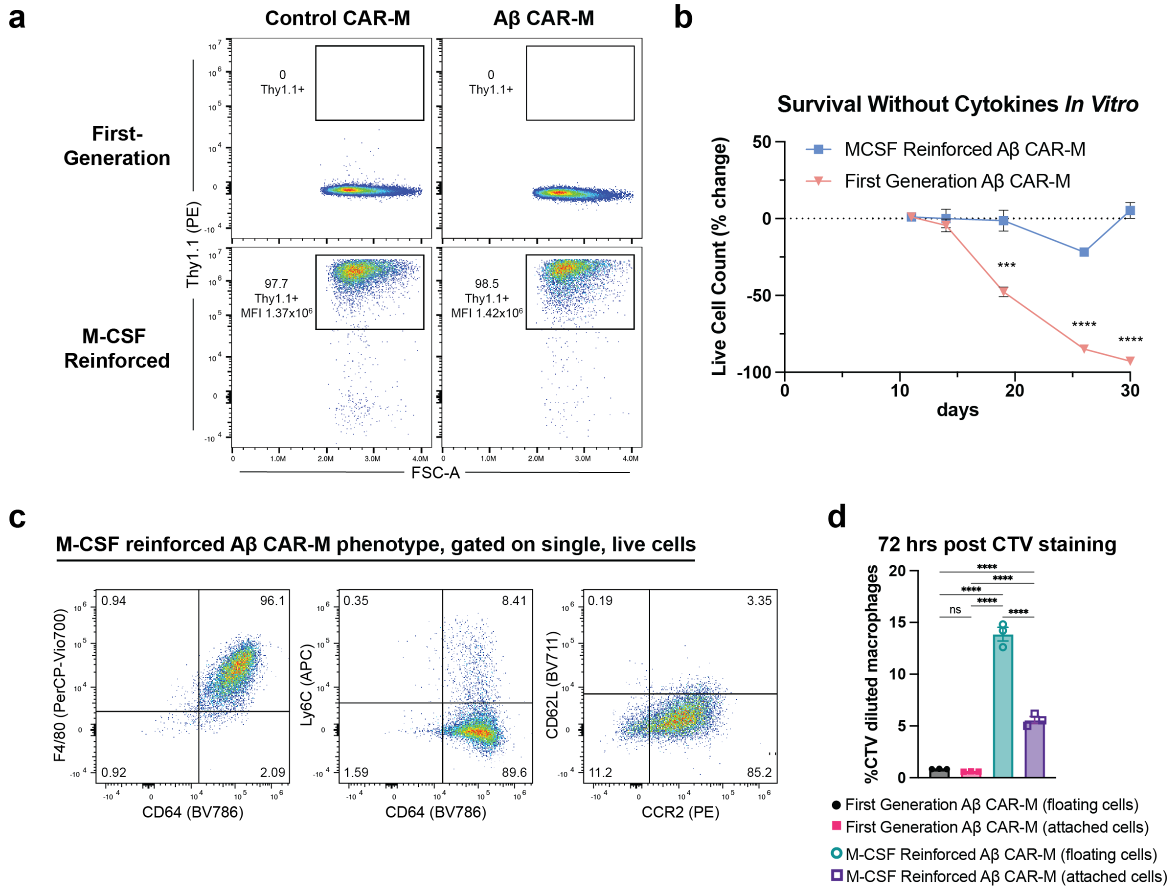

Supplemental Figure 4: Generation and characterization of M-CSF reinforced Aβ CAR-Ms. a) Representative FACS plots showing surface expression of Thy1.1 on control and Aβ CAR-Ms before and after retroviral transduction of HoxB8 cells with the M-CSF construct and sorting for Thy1.1+ cells. Gated on single, live, CAR+ cells. MFI, mean fluorescence intensity **b)** % change in live cell count of first-generation Aβ CAR-Ms and M-CSF reinforced Aβ CAR-Ms upon removal of M-CSF from the culture medium in vitro*,* determined by flow cytometry staining with ZombieNIR live/dead staining. Cells were differentiated for 6 days in M-CSF to become mature macrophages, prior to M-CSF removal. Data is representative of 2 independent experiments with 3 technical replicates per condition per experiment. Statistical significance was calculated with unpaired t-tests. **c)** Representative flow cytometry phenotyping of M-CSF

reinforced Aβ CAR-Ms after differentiating with M-CSF for 6 days in vitro. Data are representative of >3 independent experiments. **d)** Quantification of Cell Trace Violet (CTV) dilution in the stated conditions as the % of macrophages that diluted CTV 72hrs after staining. Statistical significance was calculated with a one-way ANOVA with Tukey's multiple comparisons test, and data is representative of two independent experiments with three technical replicates per condition per experiment. All data shown as mean ± s.e.m. *$P < 0.05$, **$P < 0.01$, ***$P < 0.001$, ****$P < 0.0001$, ns not significant.

supplemental 5

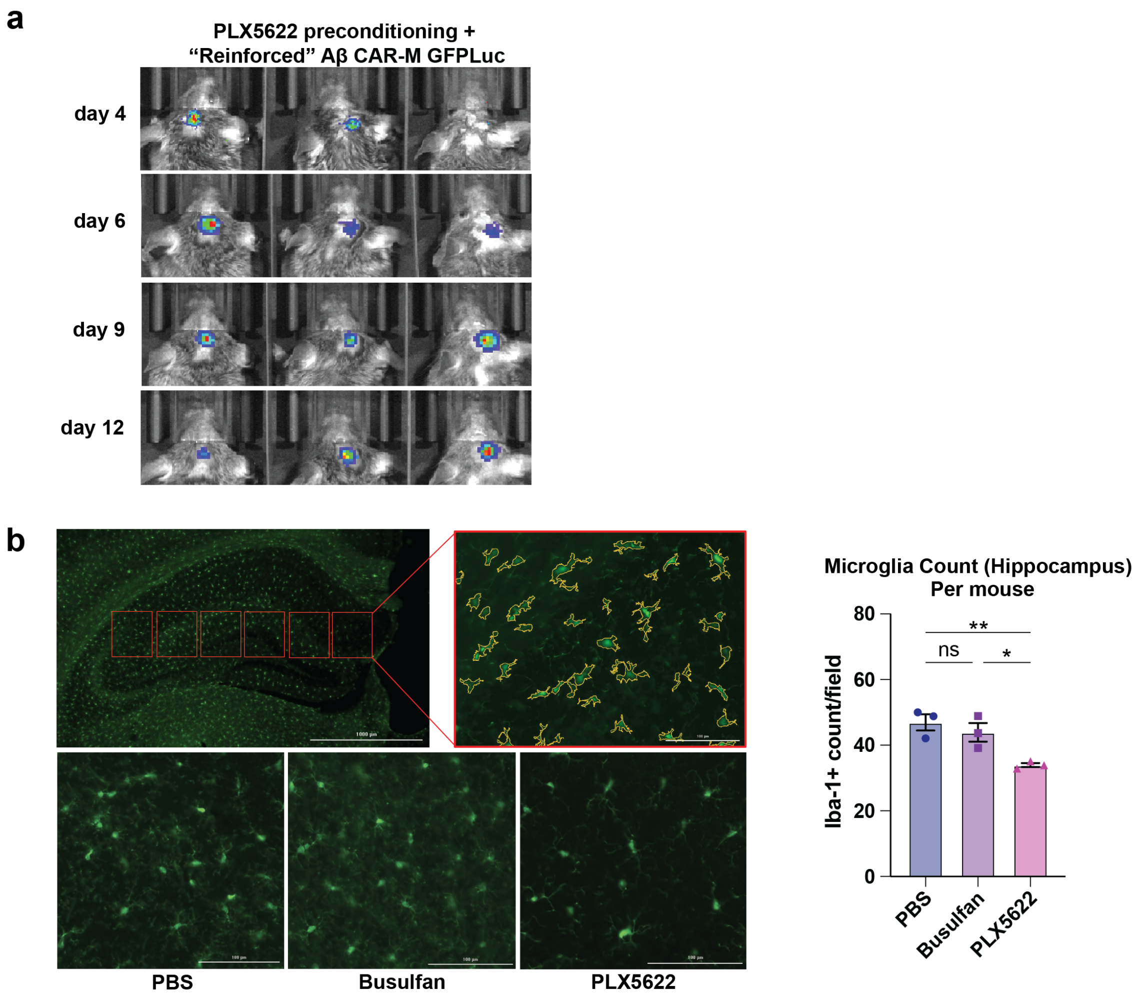

Supplemental Figure 5: M-CSF reinforced Aβ CAR-Ms expand in vivo when administered with PLX5622 preconditioning. a) Representative bioluminescence images following PLX5622 preconditioning and intrahippocampal injection of M-CSF reinforced Aβ CAR-Ms. Days indicates days post-intrahippocampal injection. **b)** (left upper) Representative image showing the six regions of interest (ROI) in which Iba-1 positive cells were quantified in each hippocampus and a higher power view of one ROI showing the cell masking used for quantification. (left lower) Representative images showing one ROI from mice treated with PBS for 2 days, 20 mg/kg Busulfan for 2 days, or 50 mg/kg PLX5622 for 4 days, twice a day. (right)

quantification of Iba-1+ cells where each dot represents the average Iba-1+ cell count in all ROIs per mouse. Statistical significance was calculated with a one-way ANOVA with Tukey's multiple comparisons test.

supplemental 6

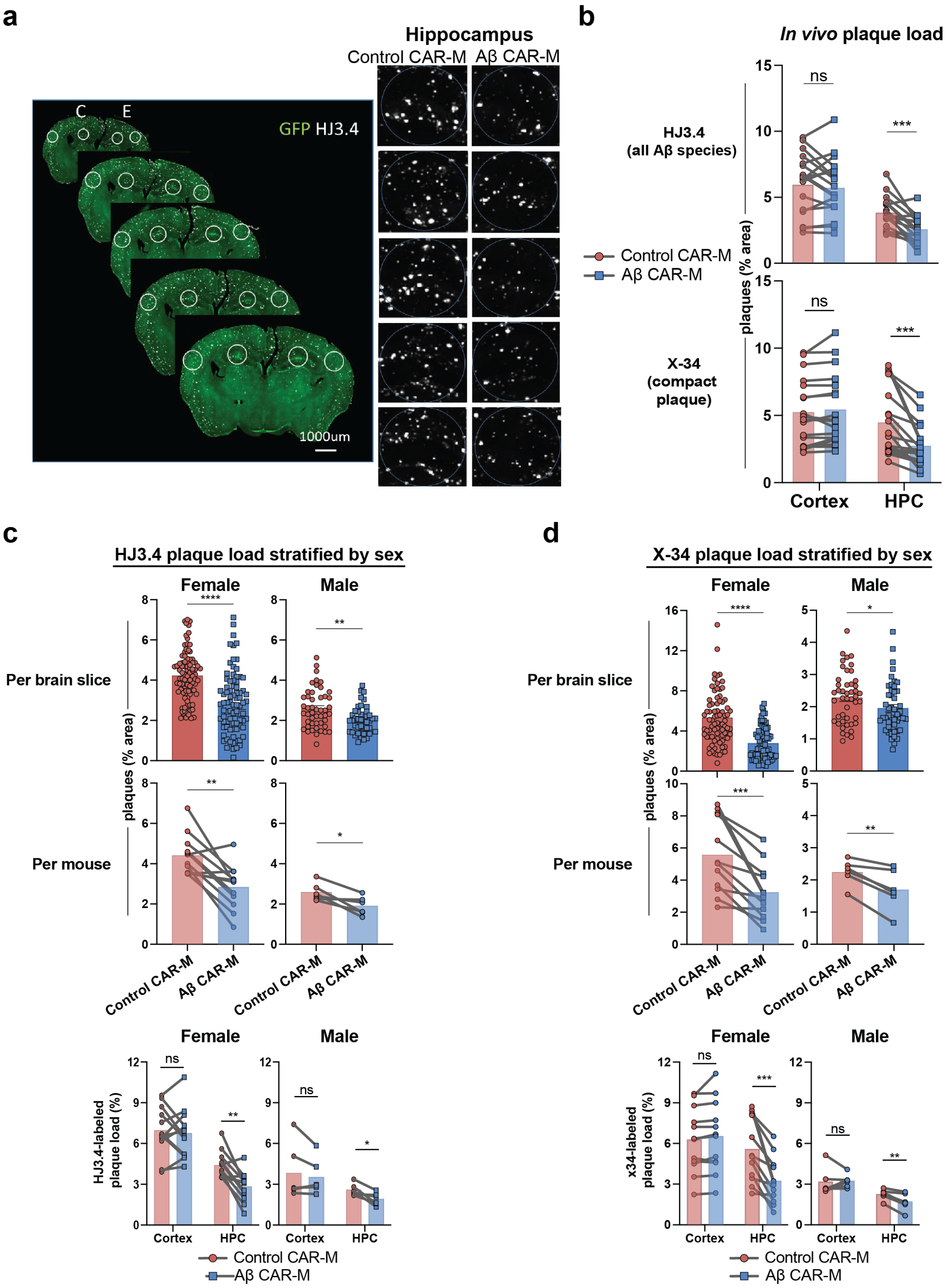

Supplemental Figure 6: M-CSF reinforced Aβ CAR-Ms reduce plaque load in the locally in the hippocampus in vivo. a) Representative images of brain sections from reinforced CAR-M treated aged APP/PS1 mice stained with HJ3.4. Images indicate circular regions of interest centered around GFP signal in the hippocampus representing the cell injection area in which plaque was quantified, highlighted in higher magnification on the right. Control regions of interest were quantified in the cortex to ensure uniform plaque load between mice. "C"= reinforced control CAR-M treated side, "E"= reinforced Aβ CAR-M treated side. **b)** Assessment of plaque load in the cortex and the hippocampus (HPC) after intrahippocampal injection of M-CSF reinforced control or Aβ CAR-Ms in n=12, 14-month-old female and n=6 13-month-old male APP/PS1 mice. Mice were sacrificed on day 12 or 13 post intrahippocampal injection and brain tissue was sectioned and stained with HJ3.4 or X-34 to assess plaque load in the regions of interest shown in **a)**. **c-d)** Plaue load data from control or Aβ CAR-M treated mice in figure 3g here stratified by sex, shown for HJ3.4 in c) and X-34 in d) per brain slice, per mouse, and comparing the cortex to the hippocampus (HPC). Data shown as mean ± s.e.m. Statistical significance was calculated with unpaired t-tests for brain slice data and paired t-tests for per mouse data. *P < 0.05, **P < 0.01, ***P < 0.001, ****P < 0.0001, ns, not significant.

Supplemental Table 1:

Antigen	Primary working concentration	Source	Secondary working concentration	Source
Lamp1	1:100	Rat anti-Lamp1 (Santa Cruz Biotechnology, cat# sc-19992)	1:800	Donkey anti-rat Cy3 (Jackson ImmunoResearch Inc., cat# 712-165-153)
Iba1	1:1000	Rabbit anti-Iba1 (Wako Pure Chemical Industries, Ltd. cat# 019-19741)	1:800	Donkey anti-rabbit Alexa Flour 488 (Thermo Fisher)
GFP	1:500	Goat anti-GFP (Rockland antibodies)	1:800	Anti-goat Alexa Flour 488 (Life Sciences)
Aβ (HJ3.4)	1:1000	Biotinylated antibody, gift from Dr. David M. Holtzman	1:800	Cy5 Streptavidin (Jackson ImmunoResearch Inc.)
